# Biological, antimicrobial, and apical tissue dissolution effects of ambroxol hydrochloride as an irrigant for regenerative endodontics: an in vitro study

**DOI:** 10.1186/s12903-026-08297-4

**Published:** 2026-04-14

**Authors:** Letycia Accioly Simoes Coelho, Ester Alves Ferreira Bordini, Fernanda Balestrero Cassiano, Mirela Cesar Barros, Renato Menezes Silva, Flaviana Bombarda de Andrade, Diana Gabriela Soares, Marco Antonio Hungaro Duarte

**Affiliations:** 1 Department of Operative Dentistry, Endodontics and Dental Materials, Bauru School of Dentistry, University of São Paulo, Bauru, SP Brazil; 2 Present Address: Present address: Department of Dental Materials and Prosthesis, Ribeirão Preto School of Dentistry, University of São Paulo, Ribeirão Preto, SP Brazil; 3 Department of Endodontics, School of Dental Medicine, University of Pittsburgh, Pittsburgh, PA USA; 4 Departament of Dentistry, Endodontics and Dental Materials, University of São Paulo, Al. Octávio Pinheiro Brizolla 9–75, Vila Universitária, Bauru, SP17012-901 Brazil

**Keywords:** Regenerative Endodontics, Stem Cells from Apical Papilla, Root canal irrigation, Antimicrobial activity, Biofilm

## Abstract

**Background:**

This study evaluated, in vitro, the effects of ambroxol hydrochloride (ABX) and sodium hypochlorite (NaOCl), alone or in combination with EDTA and activation methods on cytotoxicity and metabolic activity of stem cells from the apical papilla (SCAPs), antimicrobial, antibiofilm, and apical tissue dissolution activity.

**Methods:**

Bovine dentin specimens were used to assess SCAP viability scores (Live/Dead assay) and metabolic activity (Alamar Blue assay) in a 3D culture model, and to evaluate biofilm disruption and intratubular bacterial reduction using dual-species biofilms of *Enterococcus faecalis* and *Streptococcus mutans*. Apical tissue dissolution was assessed using bovine muscle fragments. For all experiments, specimens were allocated according to the irrigation protocol (NaOCl, ABX, or their combinations with EDTA) and further subdivided by activation method: conventional irrigation (CI), ultrasonic activation (UA), or diode laser activation (LA).

Data were analyzed with two-or three-way ANOVA and Tukey’s post hoc test (*p*<0.05).

**Results:**

NaOCl and NaOCl+EDTA showed the highest cytotoxicity scores (*p*<0.05), while ABX-based protocols demonstrated lower cytotoxicity scores and preserved SCAP metabolism (*p*<0.05). NaOCl+EDTA promoted greater biofilm reduction under CI (*p*<0.05), with no significant differences between the activation methods (*p*>0.05). In intratubular analysis, NaOCl+EDTA achieved the greatest bacterial killing, especially in the apical and superficial dentin (*p*<0.05), with LA being superior to UA (*p*<0.05). Activation improved the antimicrobial effect of ABX in superficial dentin (*p*<0.05), whereas no statistically significant differences were observed in deep dentin (*p*>0.05). For tissue dissolution, NaOCl and NaOCl+EDTA promoted significantly greater apical tissue loss across all subdivisions (*p*<0.05).

**Conclusions:**

The toxicity of the irrigating solution was the main factor influencing cell viability and metabolism, while activation impacted antimicrobial outcomes depending on the irrigant used and the dentin region. NaOCl+EDTA showed the most potent antimicrobial effects, but with higher cytotoxicity and apical tissue dissolution. ABX-based protocols were more biocompatible and, when combined with EDTA and activation, provided satisfactory antimicrobial activity, supporting ABX as a promising candidate for regenerative protocols.

**Supplementary Information:**

The online version contains supplementary material available at 10.1186/s12903-026-08297-4.

## Introduction

Regenerative endodontic procedures are a promise treatment indicate for immature permanent teeth with pulp necrosis that aim to physiologically replace or restore damaged dental tissues and the function of the pulp-dentin complex [1]. A critical step is achieving effective disinfection of the pulp space [2]. However, in immature teeth, the fragility of dentinal walls limits instrumentation, making chemical irrigation the main strategy for canal debridement [3].

Currently, the combined use of low-concentration sodium hypochlorite (1–3%) and 17% EDTA is recommended in regenerative protocols [4, 5]. NaOCl is known for its potent antimicrobial and tissue-dissolving properties [6]. However, even at low concentrations, it may compromise stem cell adhesion, proliferation, and differentiation, as well as denature growth factors [7]. To mitigate these effects, the EDTA is used as it demineralizes dentin and releases growth factors, thereby stimulating cell migration, proliferation, and differentiation [8]. However, its antimicrobial action is limited, and its capacity to reverse the cytotoxicity of NaOCl is only partial [8]. Therefore, the search for alternative solutions that better balance effective disinfection and adequate biological compatibility is necessary [9].

Ambroxol hydrochloride (ABX), widely employed in respiratory medicine [10], presents several mechanisms potentially relevant to endodontic irrigation. It exhibits antibacterial and antibiofilm activity by reducing bacterial adherence, disrupting cell-to-cell associations, downregulating genes involved in biofilm formation and efflux regulation, and interfering with quorum sensing [11]. These effects are closely related to its mucolytic action, which targets the structural integrity of the polysaccharide-rich biofilm matrix [12, 13] by decreasing its viscosity and cohesiveness [14], thereby facilitating irrigant penetration. Because elevated levels of reactive oxygen species can disrupt cellular balance and impair stem cell viability [15], the reported anti‑inflammatory and antioxidant properties of ABX [16] provided the rationale for its evaluation in the present study.

In endodontics, ABX has been tested as a vehicle for tricalcium silicate–based sealers and calcium hydroxide intracanal medicaments, enhancing their antimicrobial activity against *Enterococcus faecalis* [17, 18]. Although cytotoxic responses have been reported in fibroblasts [18], they appear to be dose- and cell type-dependent [19]. Its potential as an irrigant solution in regenerative procedures, however, remains unexplored.

To enhance the effectiveness of irrigants, activation techniques such as passive ultrasonic activation (UA) and diode laser activation (LA) have been introduced into clinical practice [9]. UA generates cavitation and acoustic streaming, improving penetration [8], while diode lasers (808–980 nm) create micro-agitation that facilitates irrigant distribution in dentinal tubules [20, 21]. Although activation enhances disinfection, concerns remain about irrigant extrusion, especially with NaOCl in immature teeth [22]. In this context, apical tissue preservation is critical because the apical papilla and its stem cells (SCAPs) represent the principal reservoir of progenitor cells that contribute to root development and dentin formation. Damage to this region may reduce the availability of these cells and compromise the biological basis of regenerative outcomes [4]. Therefore, assessing the biological impact of irrigants on this tissue is essential.

Based on this, the present study aimed to reproduce key biological conditions of immature teeth with open apices and to evaluate, in vitro, the cytotoxicity and metabolic activity of SCAPs, as well as the antimicrobial, antibiofilm, and apical tissue dissolution properties of ambroxol hydrochloride (ABX) and sodium hypochlorite (NaOCl), used alone or in combination with EDTA, under conventional irrigation (CI) or activated by ultrasound (UA) or pulsed laser (LA). The null hypothesis was that ABX would show no significant differences compared with conventional irrigants used in regenerative endodontic procedures.

## Materials and methods

### Study design

This was an in vitro experimental study conducted to evaluate the effects of different irrigation protocols on cell viability, biofilm reduction, bacterial penetration into dentinal tubules and apical tissue dissolution. The experiment was structured into five experimental groups, each with three subdivisions based on the variable under investigation. For the cell-based analyses, a factorial design was employed with three fixed factors: experimental group (5 levels), subdivision (3 levels), and time (2 intervals: days 1–3 and days 3–7), in a longitudinal model with assessments on days 1, 3, and 7. In contrast, microbiological analyses of biofilm, intratubular penetration and apical tissue dissolution were performed at a single time point. For these analyses, a two-factor factorial design (experimental group and subdivision).

### Sample size calculation

The Wilcoxon-Mann-Whitney test from the *t* test family was used. The effect size was set at 1.20 based on data from previous studies [23–25]. An alpha error of 0.05, a statistical power (beta) of 0.80, and an allocation ratio (N2/N1) of 1 were considered. The calculation indicated the need for 6 specimens per group for cell-based analyses and 8 specimens per group for microbiological procedures and apical tissue dissolution assessment. (G*Power; Heinrich Heine University, Düsseldorf, Germany).

### Group allocation

This study was approved by the Animal Research Ethics Committee (protocol number: 008/2023) and the Human Research Ethics Committee (protocol number: 7.632.860) of the Bauru School of Dentistry, University of São Paulo, Bauru, Brazil. Human samples consisted of apical papilla stem cells obtained from extracted third molars indicated for extraction as part of routine clinical care, and informed consent was obtained from the donor prior to tooth extraction. Bovine teeth were obtained from a licensed commercial slaughterhouse as post-mortem biological material following routine food industry procedures, in accordance with the approved CEUA protocol. No animals were sacrificed specifically for this study. All procedures were conducted in accordance with the guidelines of the International Organization for Standardization [26, 27].

A preliminary study was performed to determine the minimum bactericidal concentration of ambroxol hydrochloride (ABX). The lowest effective concentration identified was 3.125 mg/mL, which was adopted for this study. (Supplementary Material 1). The ABX solution was freshly prepared in distilled water before use in an amber container and protected from light and humidity at room temperature. The pH was measured immediately before use and ranged between 5 and 6.

Specimens were distributed into five experimental groups based on the irrigation protocol: Control (positive control, no treatment); NaOCl (1% sodium hypochlorite); ABX (3.125 mg/mL ambroxol hydrochloride), NaOCl + EDTA and ABX + EDTA. Each group was further subdivided according to the irrigation activation method:

- *CI (conventional irrigation)*: a 5 mL syringe (Ultradent Products Inc., South Jordan, UT, USA) with a 30G side-vented needle (MK Life, Porto Alegre, Brazil) was used and positioned 2 mm short of the working length. A aspiration tip (Capillary tips, Ultradent Products Inc., South Jordan, UT, USA) was positioned 1 mm short of the working length to generate a simulated negative-pressure.
- *UA (ultrasonic activation)*: performed with an ultrasonic device (Acteon Satelec, Paris, France) and an Irrisonic E1 tip (Helse Ultrasonic, Santa Rosa do Viterbo, Brazil) and operated at 10% power, applied in three 30-second cycles with solution renewal between cycles.
- *LA (laser activation)*: performed as in the UA group, replacing ultrasound with a diode laser (Thera Lase Surgery, DMC, São Paulo, Brazil), operating at 980 nm in pulsed mode with a power output of 1.0 W. The fiber tip was positioned 3 mm short of the apical foramen and moved spirally toward the cervical third, using upward and downward strokes.

A total of 40 mL of the primary irrigant was used in all groups, with an additional 5mL in those receiving EDTA as a secondary solution, which remained in the canal for 5 min. The average irrigation flow rate was 4 mL/min. All protocols were completed with a final rinse of 5mL sterile 0.9% saline and were performed by a single calibrated operator (endodontic specialist).

### Biological analysis

The methodological procedures followed the protocol previously described by Coelho et al., 2025 [28]. The crown and cervical portion of 180 bovine incisors were removed to obtain dentin cylinders (10 mm height) from the mid-root region to standardize the specimens. Root canals were enlarged with a Largo bur #4 (Dentsply Maillefer, Ballaigues, Switzerland), resulting in an apical diameter of 1.30 mm. The cylinders were sterilized at 121 °C for 20 min and stored in sterile distilled water at 4 °C. A primary culture of SCAPs was obtained from the apical papilla of an immature third molar (18-year-old single donor) after enzymatic dissociation with collagenase type I (3 mg/mL; Sigma-Aldrich, St. Louis, MO, USA). Informed consent was obtained from the donor prior to tooth extraction. After extraction, the teeth were coded and processed without any patient identifiers to ensure donor anonymity. The cells were characterized prior to the experiments according to the criteria described by Sonoyama et al. (2008) [29].

SCAPs up to passage 6 were cultured in α-MEM (Gibco, Invitrogen, Carlsbad, CA, USA) supplemented with 10% fetal bovine serum, L-glutamine, and 1% penicillin–streptomycin. All procedures were performed under strict aseptic conditions and regular quality control monitoring for bacterial, fungal, and mycoplasma contamination was performed during cell culture expansion.

For preparation of the three-dimensional (3D) culture, SCAPs (1 × 10⁵cells/400µL per well) were embedded in type I collagen (Collagen I, Rat Tail, Corning, NY, USA) dissolved in 10 × α-MEM (Gibco-Invitrogen, San Diego, CA, USA) at a 4:1 ratio, with pH neutralization using 5 M sodium hydroxide. Cultures were prepared directly in 48-well plate kept at 4 °C to prevent premature collagen gelation.

After collagen gelation, dentin cylinders were positioned within the culture, fixed using ethyl cyanoacrylate adhesive (Super Bonder, Loctite, Itapevi, SP, Brazil), and 300µL of supplemented α-MEM medium was added. Samples were incubated for 48 h to allow cell growth before irrigation protocols were applied. Irrigation was performed with the plate inclined at 25° to simulate clinical conditions. Scaffold morphology and structural integrity were visually monitored throughout the 7-day period, and no collapse or fragmentation was observed.

#### Cytotoxicity assessment

Cytotoxicity was evaluated on days 1, 3, and 7 (*n* = 6) using the Live/Dead Viability/Cytotoxicity kit (Invitrogen, Carlsbad, CA, USA). After removal of the dentin cylinders, 3D cultures were washed with PBS, stained for 10 min at room temperature, and transferred to glass slides for fluorescence microscopy. Four images from different fields were obtained per specimen (EVOS FL Imaging System, Invitrogen, Carlsbad, CA, USA) with standard filter sets for calcein-AM (495–515 nm) and ethidium homodimer-1 (528–617 nm). The images were coded, and qualitatively analyzed by two calibrated, blinded examiners according to a predefined scoring system in which higher scores indicate greater cytotoxic damage: Grade 0 (no cytotoxicity – preserved morphology and normal growth); Grade 1 (mild, ≤ 20% altered cells or reduced adhesion); Grade 2 (moderate, ≤ 50% altered or lysed cells); Grade 3 (moderate–severe, ≤ 70% rounded or lysed cells with > 50% growth inhibition); Grade 4 (severe, near or complete destruction of the cell layer) [26].

#### Metabolic activity assessment

Cell metabolism and proliferation were quantified using the Alamar Blue assay (Life Technologies, Carlsbad, CA, USA) on days 1, 3, and 7 (*n* = 6). At each time point, the 3D cultures were incubated with Alamar Blue reagent (10:1 ratio of serum-free α-MEM to reagent) for 4 h at 37 °C and 5% CO₂. After incubation, 100µL of the supernatant was transferred to a 96-well plate, and fluorescence was measured at 540 nm excitation and 590 nm emission (Synergy H1, BioTek, Winooski, VT, USA). The 3D cultures were then rinsed and incubated with fresh medium for subsequent time points. All analyses were performed in triplicate. Metabolic activity was expressed as the percentage change relative to the untreated control of the corresponding day, which served as the daily normalization reference.

### Microbiological analysis

All microbiological procedures were performed under aseptic conditions in a laminar flow chamber (VecoFlow Ltda, Campinas, Brazil). *Enterococcus faecalis* ATCC 29,212 and *Streptococcus mutans* ATCC 10,449 (American Type Culture Collection) were reactivated in BHI medium (Difco, Detroit, MI, USA), incubated overnight at 37 °C, adjusted to McFarland standard nº0.5 (1.5 × 10⁸CFU/mL) using a spectrophotometer (Bel Photonics, Osasco, Brazil), and incubated further to reach the exponential growth phase (7 h for *E. faecalis* and 24 h for *S. mutans*).

#### Antibiofilm activity

A total of 120 bovine dentin blocks (5 × 5 × 0.7 mm) were prepared with a 4.2 mm trephine bur (Harte Surgical Instruments, Ribeirão Preto, Brazil) under copious irrigation, sterilized at 121 °C for 20 min, and stored in distilled water. Blocks were incubated in 24-well plates containing 1.8mL of sterile BHI and 0.1mL of each bacterial suspension, under anaerobic conditions at 37 °C (Q816M20, Quimis Aparelhos Científicos Ltda, Diadema, Brazil) for 14 days. To avoid nutrient depletion, the culture medium was replaced every 48 h without adding new microorganisms.

After biofilm formation, specimens were rinsed twice with sterile distilled water. For the irrigation protocols (*n* = 8), a commercially available resin tooth model (Denarte, São Paulo, Brazil) was prepared with a cavity in the cervical third using the same trephine bur employed for block preparation. Each dentin block was fitted into this cavity with the biofilm-facing surface oriented toward the root canal lumen. Two uncontaminated blocks served as negative controls, and four contaminated but untreated blocks served as positive controls. Blocks were then stained with 50µL of Live/Dead reagent (BacLight Bacterial Viability Kit L7012; Molecular Probes, Inc.) for 10 min at room temperature before confocal microscopy analysis.

#### Intratubular antibacterial activity

A total of 120 bovine dentin cylinders (10 mm length, 1.3 mm diameter) were prepared. The smear layer was removed by three sequential ultrasonic baths of 10 min each with 2.5% NaOCl, 17% EDTA, and saline. The specimens were sterilized at 121 °C for 20 min and stored in distilled water.

Contamination was induced using the centrifugation-based protocol of Andrade et al. 2015 [30, 31]. On day 1, specimens were inoculated with *S. mutans* and subjected to eight centrifugation cycles at increasing g-forces (1400 g, 2000 g, 3600 g, and 5600 g; 5 min each at 25 °C), with inoculum renewal after each cycle. On day 3, the same procedure was repeated with *E. faecalis*. After each set of eight centrifugations, an additional cycle was performed with 100µL BHI at 3600 g for 5 min. On days 2 and 4, specimens were maintained in fresh BHI and centrifuged once at 3600 g for 5 min.

Following contamination, the specimens were subjected to irrigation protocols (*n* = 8) in a closed ex vivo model. Four specimens received no treatment and served as positive controls, while two uncontaminated specimens served as negative controls. After irrigation, specimens were sectioned longitudinally using a precision cutting machine (Isomet, Buehler, IL, USA), treated with 17% EDTA for 3 min, rinsed with saline, stained with 30µL of LIVE/DEAD BacLight reagent (Invitrogen Molecular Probes, Eugene, OR, USA) for 10 min in the dark, and prepared for confocal microscopy analysis.

#### Image analysis

All specimens were examined under a confocal laser scanning microscope (Leica TCS-SPE; Leica Microsystems, Mannheim, Germany) at 40× magnification, with 1 μm step size. For antibiofilm assays, four predetermined equidistant fields were imaged per specimen (512 × 512 pixel resolution). For intratubular assays, eight fields were acquired (two cervical and two apical, at both superficial and deep dentin; 1024 × 1024 pixel resolution) using excitation/emission settings of 488/500–540 nm for SYTO 9 and 561/600–650 nm for propidium iodide. A blinded examiner analyzed all images using Leica Application Suite-Advanced Fluorescence (LAS AF, Leica Microsystems GmbH). Quantitative parameters included the percentage of viable (green) and non-viable (red) bacteria, bacterial removal, total biovolume, viable and non-viable biovolume, and surface coverage (%). Biofilm reduction was calculated by normalizing the biovolume of treated specimens to the positive control using the formula:

$$\:\%\:reduction=1-\left(biovolume\frac{treated}{control}\right)\times100$$

The percentage of dead cells was calculated as:

$$\:\%\:dead\:cells=\left(\frac{red\:signal}{red+green\:signal}\right)\times100$$

### Apical tissue dissolution

Procedures followed the protocol described by Claudino-Ribeiro et al., 2022 [25]. Five dentin cylinders (10 mm in length and 1.3 mm in diameter) were prepared, and the apical 1 mm was temporarily sealed with a light-cured resin barrier (Biodynamics, Ipiporã, Brazil). Two wax cylinders (#7; Lysanda, São Paulo, Brazil) were adapted to the apical end to create the soft-tissue chamber. The external surface of each specimen was embedded in acrylic resin (Clássico, Campo Limpo Paulista, Brazil) to stabilize the specimen during the experiment. After polymerization, both the wax and resin barrier were removed, exposing a standardized apical cavity that served as the chamber for placement of the tissue fragment and exposure to the irrigation protocols.

Bovine muscle fragments (5 × 5 mm) were prepared immediately before the experiment and stored under 100% humidity to prevent dehydration. Each fragment was weighed three times using an analytical balance (Shimadzu, Kyoto, Japan), and the mean value was recorded as the initial weight.

To standardize specimen positioning, two reference marks were placed on the external surface of the root. First, with the tooth seated in the acrylic mold (without tissue), a line was drawn tangent to the acrylic surface. The tooth was then removed, and a second line was drawn parallel and 2 mm apical to the first line. The apical cavity was then filled with the tissue fragment, and the tooth was reinserted and pressed against the tissue using a constant apical load of 25 g (Morelli, Sorocaba, Brazil) until the first mark reached the edge of the acrylic mold, ensuring consistent compaction of the tissue fragment inside the chamber.

The cervical interface was sealed with a gingival barrier to prevent irrigant leakage and maintain stability during irrigation. After the irrigation protocol was performed (*n* = 8/group), the residual tissue fragment was carefully removed from the chamber, gently dried to remove excess moisture, and weighed three times again using the same analytical balance. The mean final weight was recorded, and tissue dissolution was calculated as the difference between the initial and final weights, representing the amount of tissue dissolved during exposure to each irrigation protocol.

### Statistical analysis

Data were subjected to the Shapiro-Wilk normality test, which indicated a non-normal distribution for most variables. Nevertheless, considering the robustness of ANOVA to moderate violations of normality in balanced experimental designs, the factorial structure of the study (group × subdivision × time), the need to evaluate interaction effects among these factors, the limitations of sequential non-parametric tests for multifactorial analyses and their potential to increase Type II error through multiple comparisons, and the use of ISO cytotoxicity scores as semi-quantitative indicators of biological response supported by methodological discussions on ordinal dental data [32], two- or three-way ANOVA, depending on the experimental model, was applied, followed by Tukey’s post hoc test for multiple comparisons. The significance level was set at 5% (*p* < 0.05). For cell viability (live/dead assay), in which independent samples were analyzed at each time point, a factorial model with three fixed factors (group, subdivision, and time) was applied. In contrast, the longitudinal analysis of cell metabolism (Alamar Blue assay), in which the same cultures were evaluated over time, was performed using a repeated‑measures factorial ANOVA model, followed by Tukey’s post hoc test. Microbiological and apical tissue dissolution analyses were conducted using two‑way factorial ANOVA models (group and subdivision) (GraphPad Software, Boston, United States).

## Results

### Cell viability

**Fig. 1:**
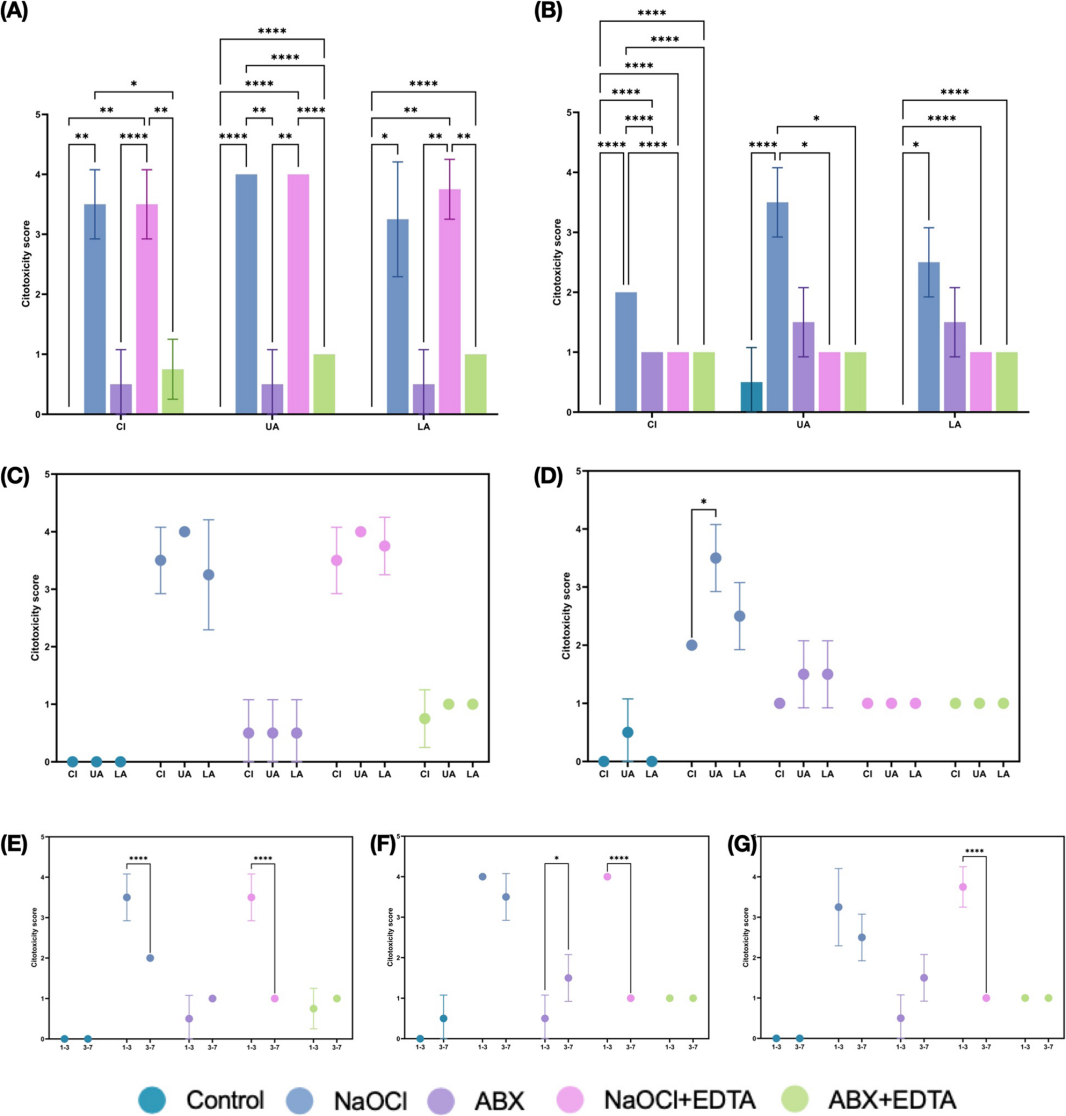
Cytotoxicity scores of SCAPs (Live/Dead assay) after exposure to different irrigation protocols. Results are shown for: comparisons among groups (Control, NaOCl, ABX, NaOCl+EDTA, ABX+EDTA) within the same subdivision at 1–3 days (**A**) and 3–7 days (**B**); comparisons between periods within the same groups at 1–3 days (**C**) and 3–7 days (**D**); and comparisons between periods for the same groups under different activation methods: CI-conventional irrigation (**E**), UA-ultrasonic activation (**F**), and LA-laser activation (**G**). Data are presented as mean ± SD. An asterisk (*) indicates statistically significant differences between groups within the same period (*p* < 0.05, three-way ANOVA and Tukey’s test)

Figure 1 presents the cell viability scores for all irrigation protocols between days 1–3 and 3–7. Within the 1–3 day period, NaOCl and NaOCl+EDTA showed the highest viability scores across all activation protocols compared to the other groups (*p* < 0.05). In contrast, ABX and ABX+EDTA maintained consistently lower values (*p* < 0.05). From 3 to 7 days, a general reduction in viability was observed (highest scores), most notably for NaOCl+EDTA, whereas NaOCl remained the group with the highest scores across subdivisions (*p* < 0.05).

Regarding comparisons among subdivisions within each group and time point, most protocols showed no significant differences (*p* > 0.05), except the NaOCl group in the 3–7 period that exhibited lower viability in the UA subdivision compared to CI and LA (*p* < 0.05).

When comparing the two time points, a significant reduction in cell viability was observed for the NaOCl+EDTA group across all subdivisions (CI, UA, and LA) (*p* < 0.05), indicating a delayed cytotoxic effect over time. NaOCl also demonstrated a significant decrease between the periods in CI while ABX demonstrated an increase between the periods (*p* < 0.05).

**Fig. 2:**
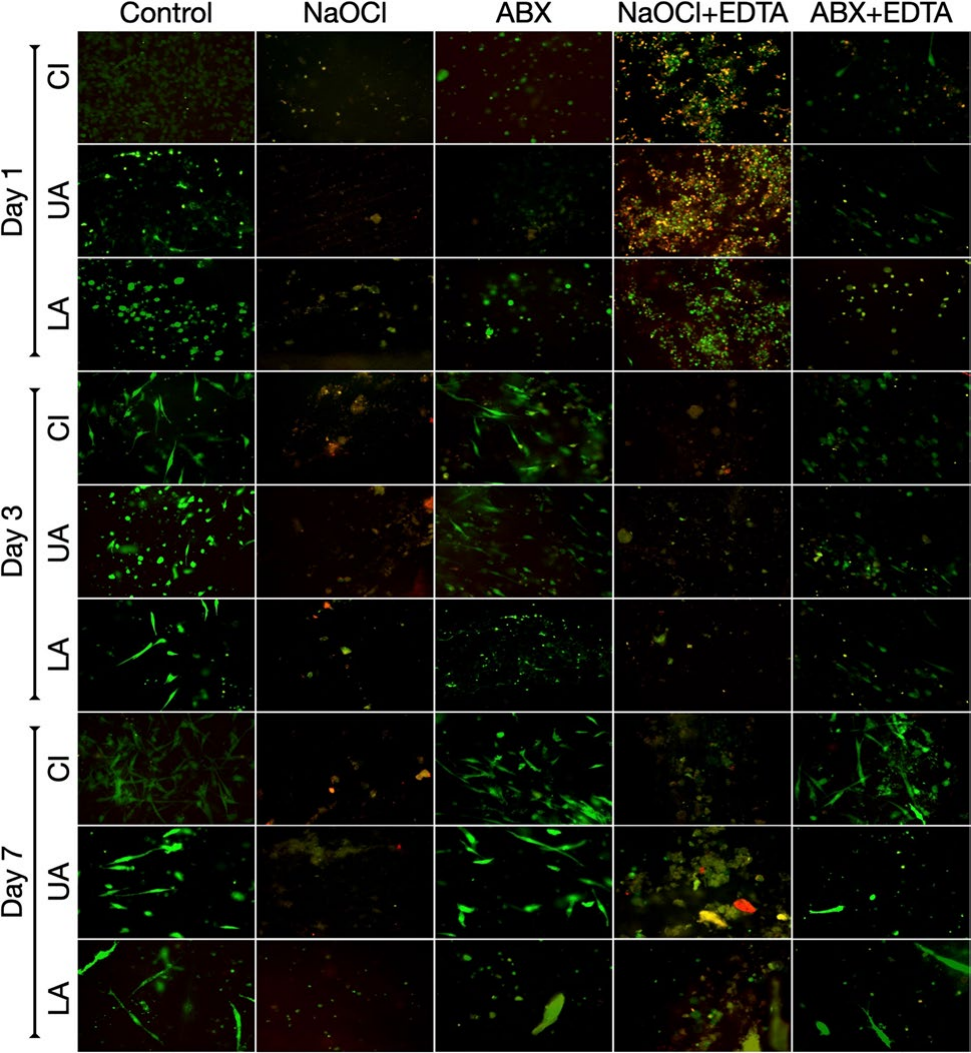
Representative fluorescence micrographs of three-dimensional (3D) SCAP cultures obtained 1, 3, and 7 days after application of the irrigation protocols. Columns represent the experimental groups (Control, NaOCl, ABX, NaOCl+EDTA, and ABX+EDTA), and rows represent the experimental conditions (CI, UA, and LA) evaluated at each time point. Green fluorescence indicates viable cells, whereas red fluorescence indicates non-viable cells

Figure 2 qualitatively illustrates cell viability on days 1, 3, and 7 after the irrigation protocols.

### Cell metabolism

**Fig. 3:**
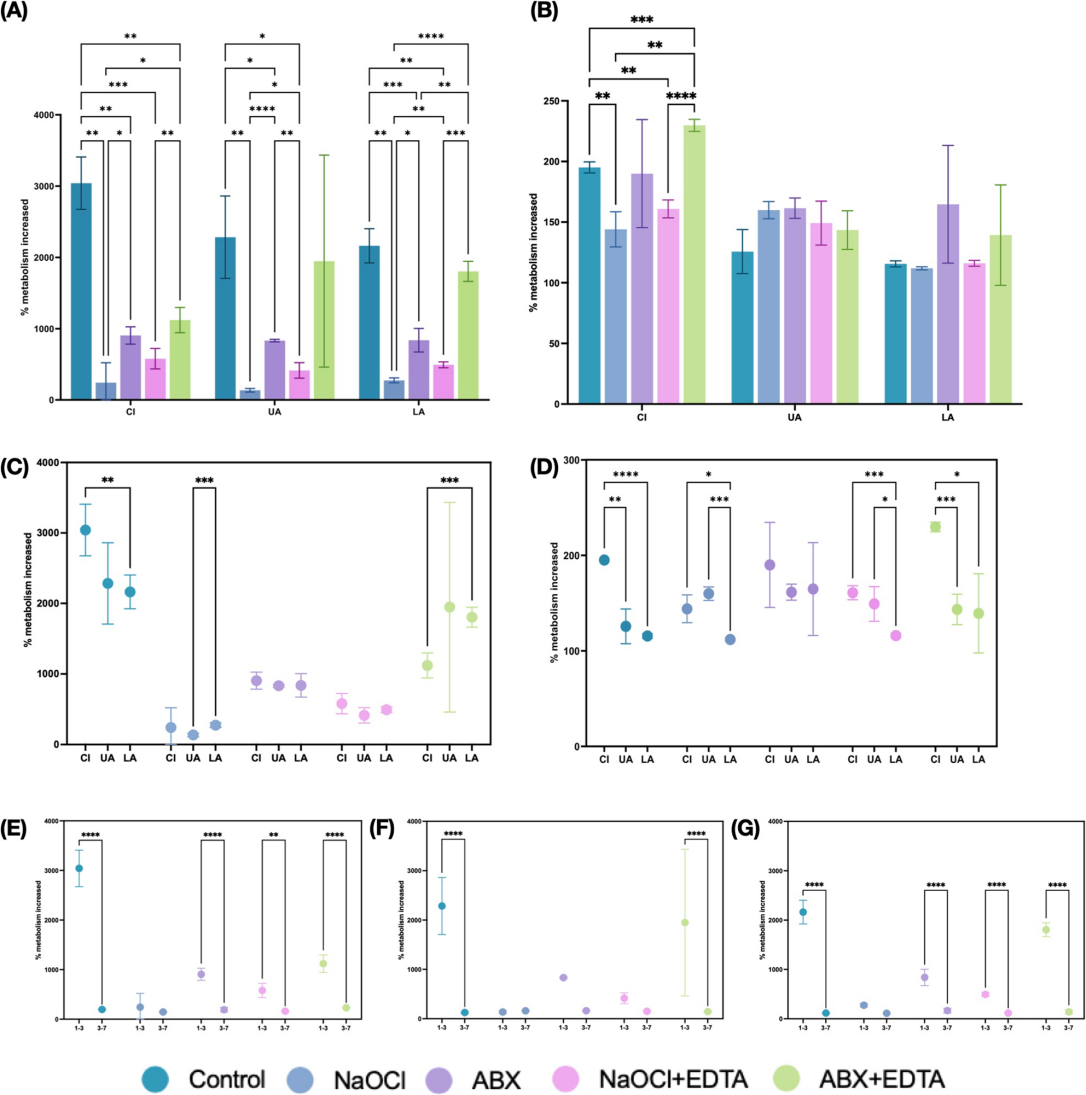
Percentage variation in metabolic activity of SCAPs (Alamar Blue assay) after exposure to different irrigation protocols. Results are shown for: comparisons among groups (Control, NaOCl, ABX, NaOCl+EDTA, ABX+EDTA) within the same subdivision at 1–3 days (**A**) and 3–7 days (**B**); comparisons between periods within the same groups at 1–3 days (**C**) and 3–7 days (**D**); and comparisons between periods for the same groups under different activation methods: CI-conventional irrigation (**E**), UA-ultrasonic activation (**F**), and LA-laser activation (**G**). An asterisk (*) indicates statistically significant differences between groups within the same period (*p* < 0.05, three-way ANOVA and Tukey’s test)

Figure 3 presents the percentage variation in cellular metabolism between days 1–3 and 3–7 for all irrigation protocols. From day 1–3, the control group exhibited the highest metabolic activity compared to all other groups (*p* < 0.05), except for ABX+EDTA in UA and LA, where no significant differences were observed (*p* > 0.05). NaOCl showed the lowest values across all subdivisions (*p* < 0.05), while ABX and ABX+EDTA were consistently higher than NaOCl (*p* < 0.05). The addition of EDTA to either NaOCl or ABX resulted in significantly higher metabolic values during the 1–3 day period compared with the use of these solutions alone (*p* < 0.05). Between days 3–7, significant differences were detected only under CI, where ABX+EDTA exhibited the highest metabolic activity compared with all other groups (*p* < 0.05).

When subdivisions were compared within the same group, in the 1–3 period, the control and ABX+EDTA groups in CI showed higher values than in LA (*p* < 0.05), and for NaOCl, LA was superior to UA (*p* < 0.05). In the 3–7 day period, both Control and ABX+EDTA maintained higher metabolism in CI compared to UA and LA (*p* < 0.05), while NaOCl-based groups showed the lowest activity under LA compared to CI and UA (*p* < 0.05).

In the within-group comparison over time, a marked reduction in metabolic activity was observed in the Control and ABX+EDTA groups across all subdivisions (CI, UA, and LA) (*p* < 0.05). ABX and NaOCl+EDTA also showed significant reductions in CI and LA (*p* < 0.05), whereas no statistically significant changes were observed in NaOCl (*p* > 0.05).

### Antibiofiolm action

**Fig. 4:**
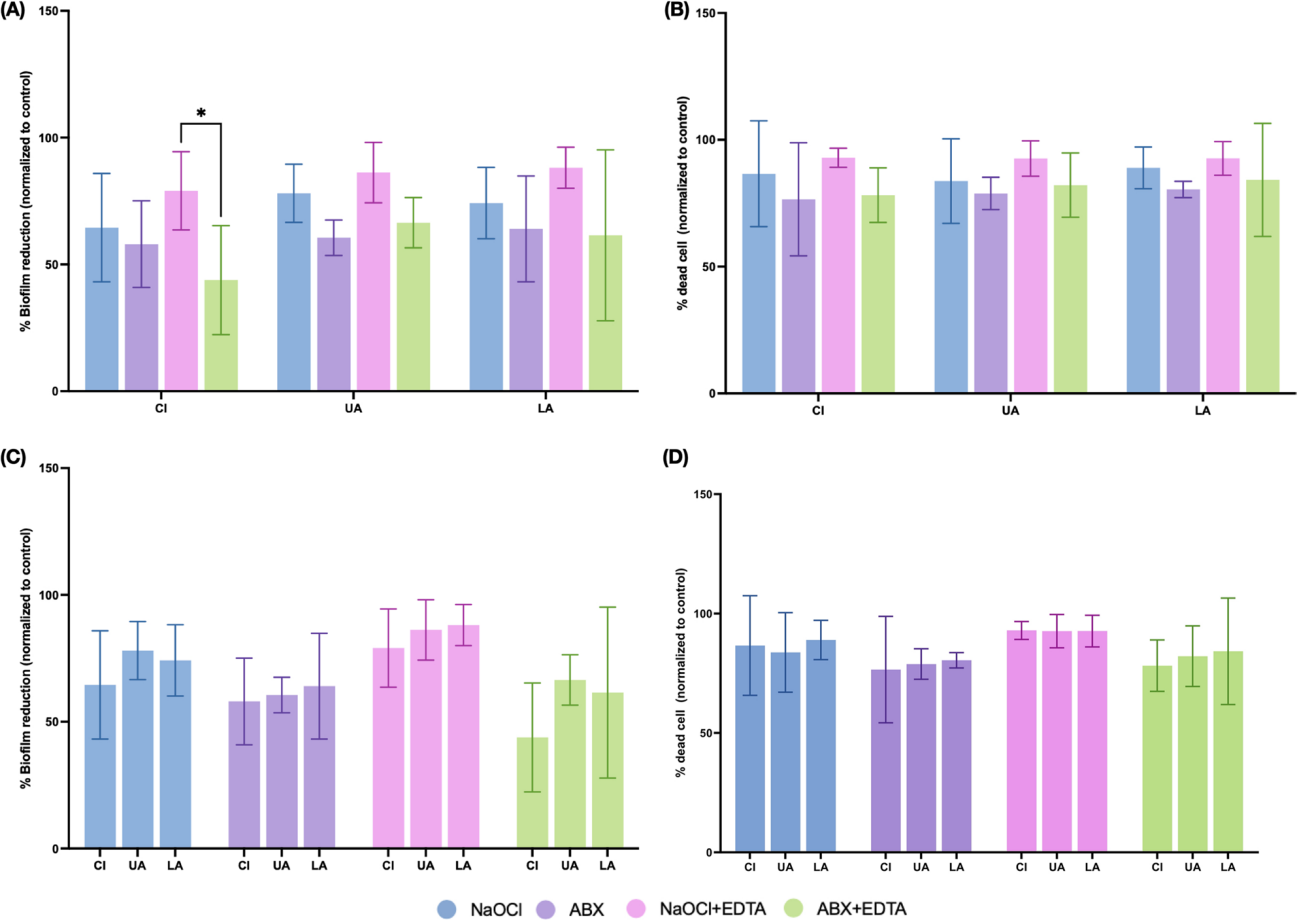
Effects of irrigation protocols on a dual-species biofilm of *E. faecalis* and *S. mutans.* Results are shown for: (**A**) percentage of biofilm reduction and (**B**) percentage of dead cells, comparing irrigation protocols (Control, NaOCl, ABX, NaOCl+EDTA, ABX+EDTA); and (**C**) percentage of biofilm reduction and (**D**) percentage of dead cells, comparing activation methods (CI-conventional irrigation, UA-ultrasonic activation, LA-laser activation). Data are expressed as mean ± SD. An asterisk (*) indicates statistically significant differences compared with the control group (*p* < 0.05, two-way ANOVA with Tukey’s test)

**Fig. 5:**
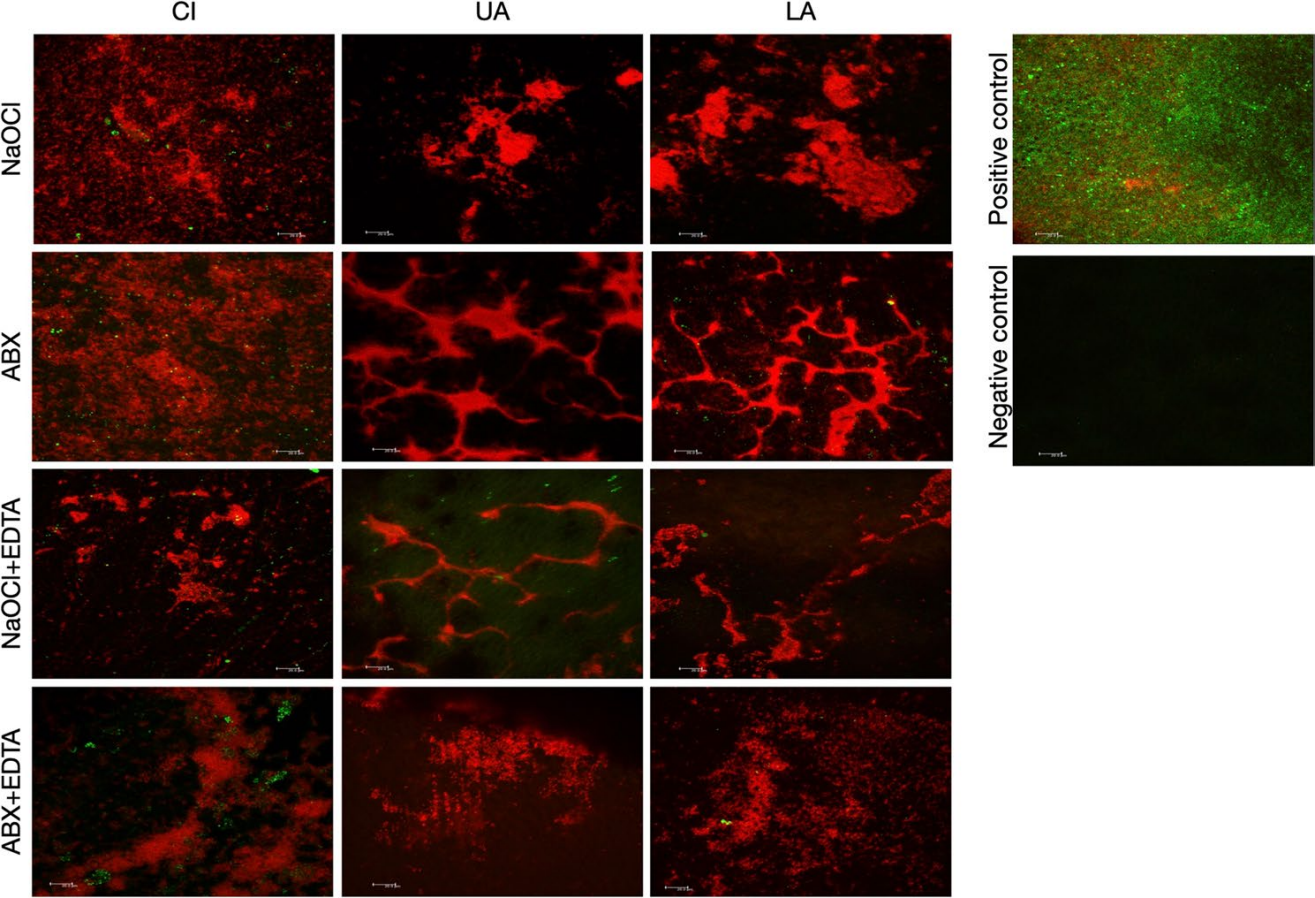
Representative confocal laser scanning microscopy images of dual-species E. faecalis and S. mutans biofilms after application of the irrigation protocols under the three experimental conditions (CI, UA, and LA). Images were obtained by confocal microscopy with Z‑stack reconstruction to visualize biofilm architecture for each treatment group (NaOCl, ABX, NaOCl+EDTA, and ABX+EDTA), with the untreated positive control included for comparison. Viable bacteria are shown in green and non‑viable bacteria are shown in red. Original magnification ×40; scale bars = 20 μm

Figure 4 shows the effects of the irrigation protocols on biofilm reduction and bacterial viability. In terms of biofilm reduction, a significant difference was observed only under CI, where NaOCl+EDTA promoted a greater reduction compared with ABX+EDTA (*p* < 0.05). When activation was applied (UA or LA), no significant differences were detected among the experimental groups, and no significant differences were observed among subdivisions (*p* > 0.05). Regarding bacterial viability, none of the protocols produced significant differences either between groups or among subdivisions (*p* > 0.05). Figure 5 qualitatively illustrates these findings, highlighting the absence of clear differences in bacterial viability among the tested protocols.

### Intratubular antibacterial action

**Fig. 6:**
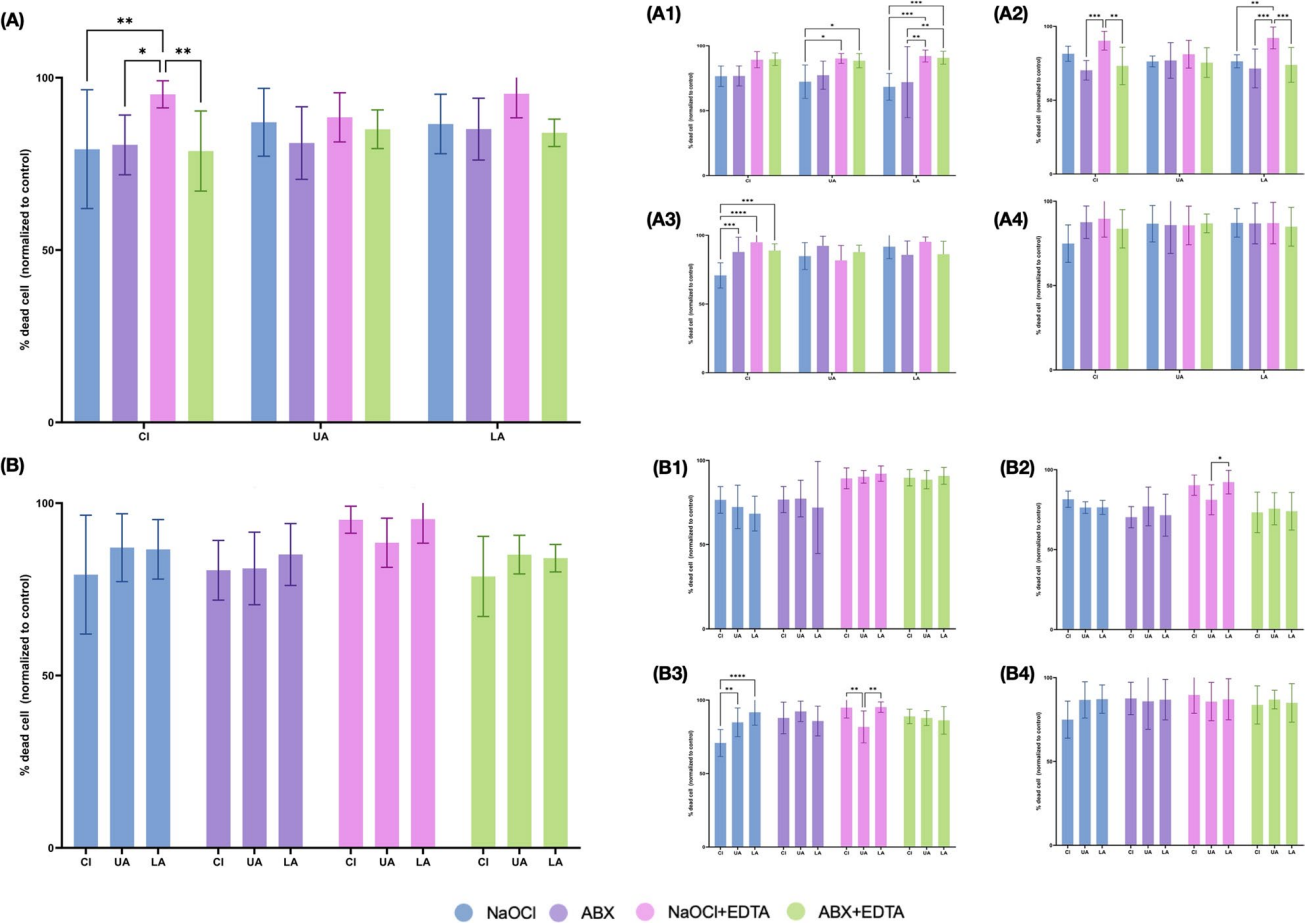
Percentage of dead bacteria in dentinal tubules after irrigation with different protocols (NaOCl, ABX, NaOCl+EDTA, ABX+EDTA, and Control). Results are shown for: (**A**) comparisons among groups and (**B**) comparisons among activation methods (CI-conventional irrigation, UA-ultrasonic activation, LA-laser activation) in the total analysis. Sub-analyses include (1) apical third, (2) cervical third, (3) superficial dentin, and (4) deep dentin. Data are expressed as mean ± SD. An asterisk (*) indicates statistically significant differences compared with the control group (p < 0.05, two-way ANOVA with Tukey’s test)

**Fig. 7:**
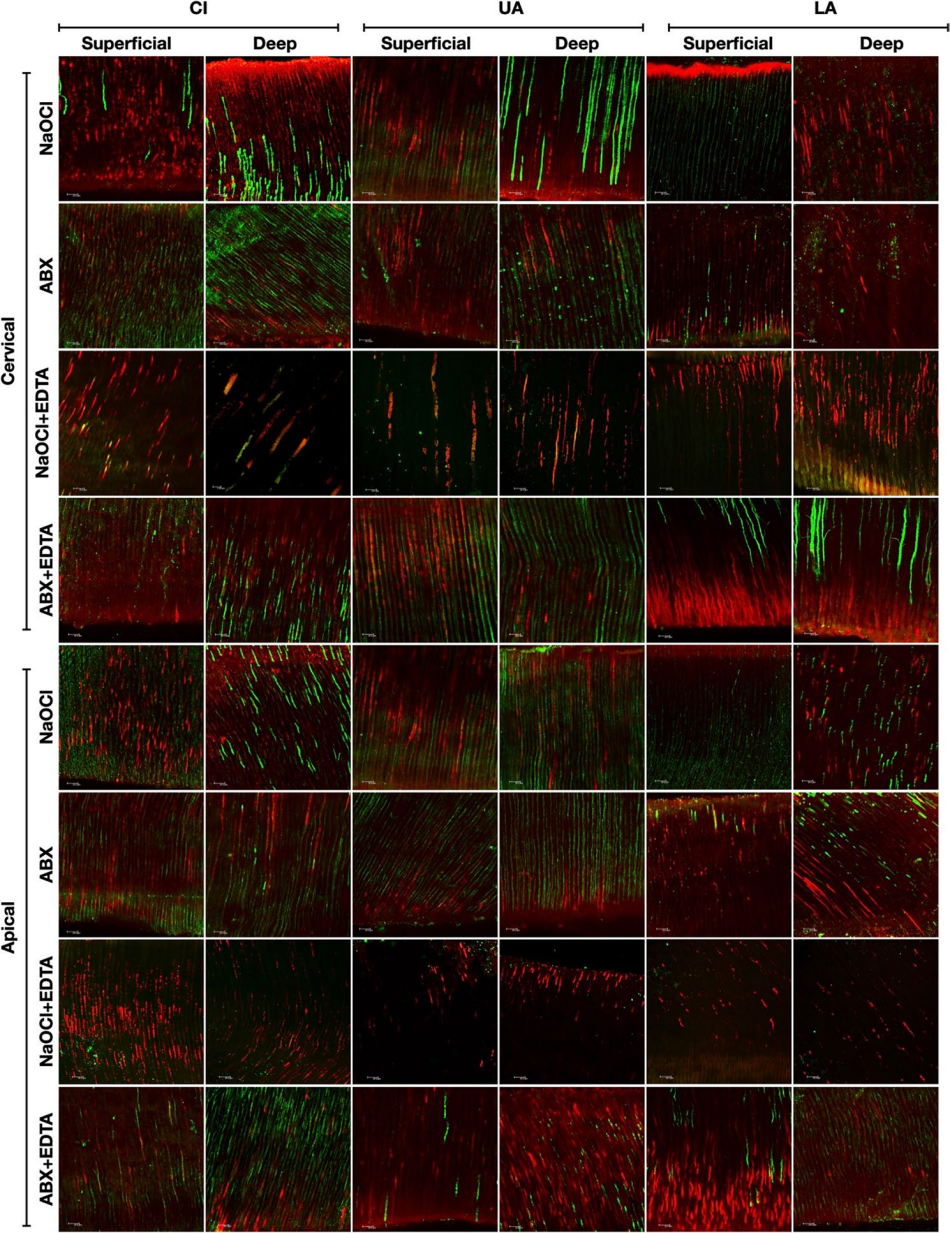
Representative confocal laser scanning microscopy images of intratubular bacterial contamination following application of the irrigation protocols. Images illustrate bacterial penetration within dentinal tubules in the cervical and apical thirds of the root canal, evaluated at superficial and deep dentin levels under the CI, UA, and LA experimental conditions for each irrigation protocol (NaOCl, ABX, NaOCl+EDTA, and ABX+EDTA). Viable bacteria are shown in green and non-viable bacteria in red. Original magnification ×40; scale bars = 20 μm

Figure 6 shows the percentage of dead bacteria in intratubular dentin after the irrigation protocols, analyzed in total, apical, cervical, superficial, and deep dentin. Figure 7 qualitatively illustrates the intratubular viability after treatment with the different protocols.

In the total analysis, the NaOCl+EDTA group under CI showed the highest percentage of dead cells compared to all other groups (*p* < 0.05), while no differences were detected under UA or LA (*p* > 0.05). In the apical third, NaOCl consistently resulted in fewer dead cells than EDTA-based protocols in UA, and it showed the lowest values overall in LA (*p* < 0.05). Similarly, in LA, ABX alone also resulted in a lower percentage of dead cells compared with ABX+EDTA (*p* < 0.05). In the cervical third, NaOCl+EDTA promoted significantly more dead cells than ABX and ABX+EDTA in CI and LA (*p* < 0.05). However, NaOCl alone under LA howed a lower percentage of dead cells compared with the other groups (*p* < 0.05). When comparing subdivisions, a difference was only observed for NaOCl+EDTA, with LA showing a higher percentage of dead cells than UA (*p* < 0.05).

In the superficial dentin, differences were restricted to CI, where NaOCl showed the lowest percentage of dead cells compared to all groups (*p* < 0.05). In CI, NaOCl showed fewer dead cells than UA and LA; while in NaOCl+EDTA, UA showed a lower percentage of dead cells compared with CI and LA (*p* < 0.05). In the deep dentin, no significant differences were observed between groups or subdivisions (*p* > 0.05).

### Tissue dissolution

**Fig. 8:**
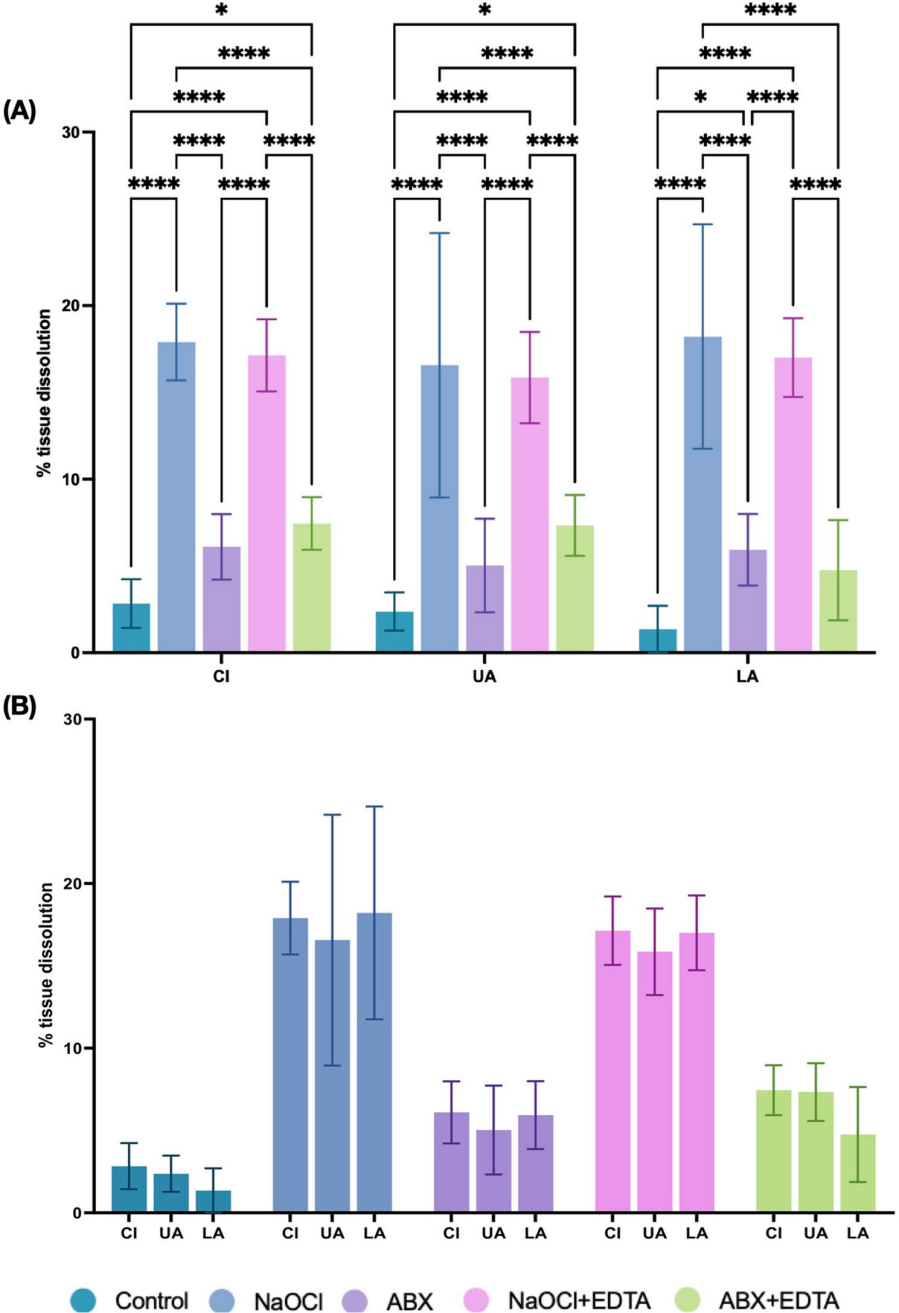
Percentage of apical tissue dissolution after irrigation protocols. Results are shown for: (**A**) comparisons among groups (NaOCl, ABX, NaOCl+EDTA, ABX+EDTA, and Control) and (**B**) comparisons among activation methods (CI-conventional irrigation, UA-ultrasonic activation, LA-laser activation). Data are expressed as mean ± SD. An asterisk (*) indicates statistically significant differences compared with the control group (*p* < 0.05, two-way ANOVA with Tukey’s test)

Figure 8 illustrates the percentage of apical tissue dissolution following the various irrigation protocols. In all subdivisions, NaOCl and NaOCl + EDTA promoted significantly greater dissolution compared to the other groups (*p* < 0.05). Within the UA and CI subdivisions, ABX+EDTA also showed higher dissolution than the control (*p* < 0.05), whereas in LA, ABX alone produced greater dissolution than the control (*p* < 0.05). No significant differences were observed among subdivisions within the same group (*p* > 0.05).

## Discussion

This study evaluated, in vitro, the cytotoxicity, metabolic activity of SCAPs, and the antimicrobial and antibiofilm properties of different irrigation protocols. The null hypothesis was rejected, as significant differences were observed across all analyses. Sodium hypochlorite, either alone or in combination with EDTA, demonstrated high antimicrobial and antibiofilm efficacy; however, it was associated with increased cytotoxicity and more apical tissue dissolution. In contrast, protocols containing ambroxol hydrochloride, whether combined with or without EDTA, exhibited a more favorable biological profile, with reduced cytotoxicity and satisfactory antimicrobial performance.

Proper selection of irrigants is crucial for regenerative procedures. Ideally, they should eradicate microorganisms, dissolve necrotic tissue and biofilm, preserve dentin, release bioactive factors, and remain biocompatible by supporting cell adhesion, proliferation, and differentiation [33]. Because the biological effects of ambroxol are concentration-dependent, particularly regarding cytotoxicity, a preliminary minimum bactericidal concentration (MBC) assay was performed to determine the lowest concentration capable of eliminating the tested microorganisms. The selected concentration (3.125 mg/mL) therefore represents a balance between antimicrobial efficacy and biological compatibility, which is a critical requirement for regenerative endodontic protocols. The activation method also influences irrigant distribution within dentinal tubules [3, 34]. Techniques such as passive ultrasonic activation (UA) and diode laser activation (LA) have been proposed [20, 21].

Nonetheless, irrigant extrusion remains a major concern because it compromises stem cell viability, which is fundamental for tissue regeneration [22, 35]. In the present study, bovine muscle fragments were used to assess apical tissue dissolution because this substrate offers high availability, consistent composition, and easier standardization of specimen dimensions [36]. Both NaOCl and NaOCl+EDTA caused significantly greater dissolution compared to the other groups (*p* < 0.05). This finding aligns with previous reports highlighting the strong tissue-dissolving capacity of NaOCl [6, 25]. Interestingly, agitation did not influence this outcome, which the wide apical diameter and the reduced resistance of apical tissues may explain. However, excessive apical tissue dissolution can reduce the availability of cells, thereby negatively impacting regenerative outcomes.

To address this, SCAPs were cultured in a 3D model using bovine dentin cylinders, thereby preserving cell–matrix interactions and more accurately replicating clinical conditions [28]. In all protocols, irrigation was performed with the plate inclined at 25°, using a side-vented needle positioned 3 mm short of the apical foramen [34]. Ultrasonic and laser activation tips were likewise maintained 3 mm from the apex to minimize extrusion risk and enhance solution distribution. Intermediate irrigation with sterile saline was applied between solutions to prevent undesirable chemical interactions and ensure proper neutralization [7, 8].

In this model, both cytotoxicity and metabolic assays were conducted to understand the SCAPs’ biological response. Cytotoxicity reflects direct effects on cell survival, while metabolic activity reveals functional capacity and recovery after irrigant exposure [37].

In the cytotoxicity analysis, NaOCl and NaOCl+EDTA showed the greatest reduction in viability between days 1–3 across all subdivisions (CI, UA, LA), compared to the ABX-based groups (*p* < 0.05). From days 3–7, NaOCl remained the most cytotoxic, while NaOCl+EDTA caused a further decline in viability, suggesting a delayed toxic effect, as previously reported in studies [8]. In contrast, ABX showed a slight increase in viability over time, suggesting a more favorable biological response.

For metabolic activity, the control group presented the highest values on days 1–3 (*p* < 0.05). During this period, ABX and ABX+EDTA consistently exhibited greater metabolic activity than NaOCl (*p* < 0.05), with EDTA further enhancing this effect, particularly under activation (UA and LA). From days 3–7, ABX+EDTA under CI maintained the highest metabolic rates among all groups (*p* < 0.05), whereas NaOCl remained the lowest across subdivisions. Over time, both control and ABX+EDTA groups showed a gradual decline in activity, while NaOCl remained consistently low throughout the evaluation period.

The divergence between cytotoxicity and metabolic results suggests that some irrigants do not cause immediate cell death but can still impair cellular function. In this study, ABX-based protocols preserved viability but showed lower metabolism than the Control in the early period (days 1–3). A similar pattern was observed with NaOCl+EDTA, where short-term viability was maintained but metabolic activity declined. These findings highlight the importance of combining cytotoxicity and metabolic assays, since functional alterations may occur even when cell survival appears unaffected.

However, this pattern was not observed in the LA subdivision. In this subgroup, ABX+EDTA maintained metabolic activity similar to the control during the early period, despite reduced viability at later time points. This finding suggests that diode laser activation at 980 nm, applied in pulsed mode at low power (1 W), may result in less metabolic impairment compared with ultrasonic activation. This effect may be related to differences in the mechanical agitation produced by the activation methods, with laser activation potentially generating less disruptive physical stress on the cells.

Although understanding biological effects is essential, antimicrobial performance must also be taken into consideration. Assessing antibiofilm activity is particularly relevant, as biofilms are the predominant microbial form in endodontic infections and are more resistant to irrigants [34, 38].

This study used a dual-species biofilm of *Enterococcus faecalis* and *Streptococcus mutans*, both commonly found in endodontic infections [39]. *E. faecalis* is highly resistant, able to survive in harsh conditions and persist inside dentinal tubules even with limited nutrients [40]. *S. mutans*, on the other hand, is associated with symptomatic canals [41] and increases biofilm thickness and complexity [38], which helps *E. faecalis* adhere and persist [39]. The interaction of these species makes the model more realistic and challenging for testing the efficacy of irrigants.

To further strengthen the model, mature biofilms (14 days) present a higher bacterial load, greater structural complexity, deeper tubule penetration, and increased resistance to antimicrobials [2, 42]. Therefore, irrigants must not only eliminate bacteria but also reduce biofilm mass and clean dentinal walls. Even after viable cells are eradicated, the residual extracellular matrix can hinder the penetration of subsequent irrigants, prevent complete disinfection, and compromise stem-cell adhesion and proliferation [42].

In this study, NaOCl+EDTA produced greater biofilm reduction under CI than ABX+EDTA (*p* < 0.05). However, with activation (UA or LA), no significant differences were found among groups or subdivisions (*p* > 0.05). For bacterial viability, all irrigants showed a similar proportion of dead cells (*p* > 0.05). Although NaOCl is recognized as the most effective irrigant against biofilms [39], the low concentration and absence of instrumentation may have reduced its efficacy.

Beyond antibiofilm effects, intratubular antimicrobial activity is critical, as microorganisms inside dentinal tubules are difficult to eliminate [43]. Clinical evidence indicates that successful revitalization depends not only on microbial reduction but also on maintaining biological conditions that support tissue regeneration [44]. In this context, stem cells from the apical papilla (SCAPs) play a critical role in root maturation and pulp-dentin regeneration because of their high proliferative capacity and differentiation potential [45]. In immature teeth, wider dentinal tubules facilitate deeper bacterial penetration [3], and the limited instrumentation characteristic of regenerative procedures increases reliance on the chemical properties of irrigants [7, 38]. Consequently, evaluating antimicrobial performance, including the ability of irrigants to act within dentinal tubules, becomes particularly relevant. At the same time, these antimicrobial effects must be interpreted together with their potential impact on SCAP survival and function, as excessive cytotoxicity may compromise regenerative outcomes. Moreover, unlike conventional therapy, regenerative procedures do not provide immediate canal obturation, leaving residual bacteria unsealed, while the apical blood supply may further support their survival [46].

In this study, NaOCl+EDTA under conventional irrigation resulted in the highest percentage of dead cells in both the total analysis and the cervical third (*p* < 0.05). In the cervical region, this superiority was also observed under LA (*p* < 0.05). In contrast, in the apical third, significant differences appeared only with activation, suggesting agitation is decisive in this area. This may be explained by the greater number and diameter of the tubules in the cervical third, which increase chemical differences among irrigants when agitation is limited. In the apical third, with fewer and narrower tubules, activation seems essential for penetration.

For dentin depth, differences in the superficial layer were observed only under CI, where NaOCl showed the lowest percentage of dead cells compared to all other groups (*p* < 0.05). Subgroup analysis confirmed that NaOCl under CI had fewer dead cells than UA and LA, while NaOCl+EDTA under UA was less effective than CI and LA (*p* < 0.05). These findings indicate that performance in superficial dentin is strongly influenced by activation, which facilitates penetration and bacterial killing near tubule entrances.

In contrast, no significant differences were detected in deep dentin. This may be due to the greater accessibility of superficial dentin, which has lower mineral density, larger tubules, and reduced curvature, thereby favoring penetration and biofilm disruption [47]. Deeper dentin, with higher mineral content and narrower tubules, restricts diffusion and may reduce the activity of chemical agents [48].

Despite the relevance of these findings, limitations of the in vitro model must be acknowledged. Laboratory conditions cannot fully reproduce clinical complexity [49], including tissue pressure, gravity, canal variability, and apical blood flow, all of which affect irrigant distribution and clearance. Amboxol hydrochloride was evaluated at a single concentration determined by a preliminary MBC assay to identify the lowest dose capable of eliminating the tested microorganisms while maintaining biological compatibility. Further dose–response studies are needed to determine its optimal therapeutic range for regenerative endodontic applications. The study also used 1% NaOCl instead of the guideline‑recommended 1.5% concentration suggested for regenerative endodontic procedures. This lower concentration was adopted to maintain conditions compatible with SCAP biological assessment while still allowing evaluation of antimicrobial and tissue‑dissolution performance within the experimental model. Even though the guideline concentration was not directly tested, the model enabled meaningful comparison among the protocols and provided relevant insight into the potential role of ABX as an alternative irrigant for regenerative endodontic strategies. Future studies should directly compare ABX with the guideline‑recommended NaOCl (1.5%)+EDTA protocol to further position these findings within current clinical recommendations.

Although the use of 3D cell cultures and bovine dentin cylinders increases clinical relevance, limitations persist, such as the absence of vascularization, immune response, and tissue remodeling [50]. Bovine dentin was used in all assays because it offers a reproducible and ethically accessible substrate with structural and chemical characteristics broadly comparable to human dentin, and its suitability has been validated for antimicrobial and biological assays [51]. Nevertheless, bovine dentin does not fully replicate the biological environment of immature human dentin, which should be considered when interpreting the findings. Additionally, the use of bovine muscle to assess tissue dissolution allows experimental standardization and has been widely used in laboratory studies; however, it does not fully reproduce the structural and biochemical characteristics of human pulp or periapical tissues. Therefore, the findings should be interpreted with caution.

Likewise, although the mature dual-species biofilm and intratubular contamination do not reproduce the full complexity of natural infections, the *E. faecalis* and *S. mutans* model provides a standardized and reproducible system for evaluating microbial interactions and antimicrobial performance [39]. However, it should be noted that CLSM with Live/Dead staining evaluates membrane integrity rather than bacterial reproductive viability. Even with these limitations, this study contributes valuable data for the development of new strategies. To our knowledge, this is the first report evaluating ambroxol hydrochloride in regenerative irrigation protocols. As an initial investigation, the present study focused on establishing the antimicrobial performance and biological compatibility of this compound under controlled in vitro conditions.

These findings provide a foundation for future investigations exploring the broader biological implications of ambroxol-based irrigation strategies. In particular, subsequent studies should evaluate the interaction of these protocols with dentin substrates and their potential influence on the release of dentin-derived growth factors. Complementary approaches, including dentin chemical analyses and molecular techniques such as gene expression assays (e.g., PCR), may help clarify the mechanisms underlying these effects. Further validation through additional experimental models and clinical studies will also be necessary to determine the translational potential of ambroxol-based irrigation protocols in regenerative endodontic therapy.

Within the limitations of this in vitro study, sodium hypochlorite, particularly in association with EDTA, demonstrated the greatest antimicrobial and antibiofilm activity; however, it was also associated with higher cytotoxicity and apical tissue dissolution. Ambroxol hydrochloride maintained SCAP viability and metabolic activity under the experimental conditions evaluated. When combined with EDTA and activation methods, it achieved antimicrobial effects without a significant increase in cytotoxicity. These findings suggest that the choice of irrigant and activation strategy influence the balance between antimicrobial efficacy and biological compatibility under the experimental conditions evaluated. Ambroxol hydrochloride may represent a candidate for further investigation in regenerative endodontic protocols.

## Supplementary Information

Supplementary Material 1.

## Data Availability

The data that support the findings of this study are available from the corresponding author, Letycia Accioly Simoes Coelho, upon reasonable request.
