# Supplementary material for: Biological, antimicrobial, and apical tissue dissolution effects of ambroxol hydrochloride as an irrigant for regenerative endodontics: an in vitro study
Source: BMC Oral Health. 2026 Apr 14;26:1012. doi: 10.1186/s12903-026-08297-4 (PMC13255274; doi:10.1186/s12903-026-08297-4)
Supplement: Supplementary file 1 — Supplementary Material 1. [file 12903_2026_8297_MOESM1_ESM.docx]

*Supplementary Material 1: Preliminary Antimicrobial Test*

Disinfection is a critical requirement for the success of regenerative procedures, necessitating the use of irrigating solutions with strong antimicrobial activity. Ambroxol Hydrochloride (ABX) is a substance widely used in medical practice and known for its antimicrobial properties. However, there are no previous reports regarding its use as a root canal irrigant or on its effective bactericidal concentration. Therefore, an antimicrobial test was conducted to determine the minimum bactericidal concentration of Ambroxol Hydrochloride (ABX) when used as an irrigating solution against *Enterococcus faecalis*.

For the assay, 3 mL of sterile Brain Heart Infusion (BHI) broth (50-488-527, Difco, Detroit, MI, USA) were added to 12 test tubes. A stock solution of ABX at 100 mg/mL was prepared, from which ten serial dilutions were made (100, 50, 25, 12.5, 6.25, 3.125, 1, 0.5, 0.25, and 0.1 mg/mL), and added to the BHI-containing tubes. One tube containing 100 mg/mL ABX was kept as a positive control.

The *Enterococcus faecalis* ATCC 29212 strain was reactivated in BHI broth and incubated at 37°C for 24 hours. The bacterial suspension was adjusted to McFarland standard No. 0.5 (1.5 × 10⁸ CFU/mL) using a spectrophotometer (Bel Photonics do Brasil Ltda., Osasco, SP, Brazil, model SF325NM), and 0.83 µL of the inoculum was added to each tube containing the various concentrations of the test solution, as well as to the negative control.

After 24 hours of contact between the solutions and the bacterial inoculum, 50 µL from each tube were plated onto Petri dishes containing BHI agar, which had been previously prepared and stored under refrigeration for 24 hours. Plates were then incubated at 37°C for 48 hours.

Qualitative analysis demonstrated that the concentration of 3.125 mg/mL was the lowest capable of completely inhibiting bacterial growth of the tested strain. Therefore, this concentration was selected for use in the experiments of the present study.


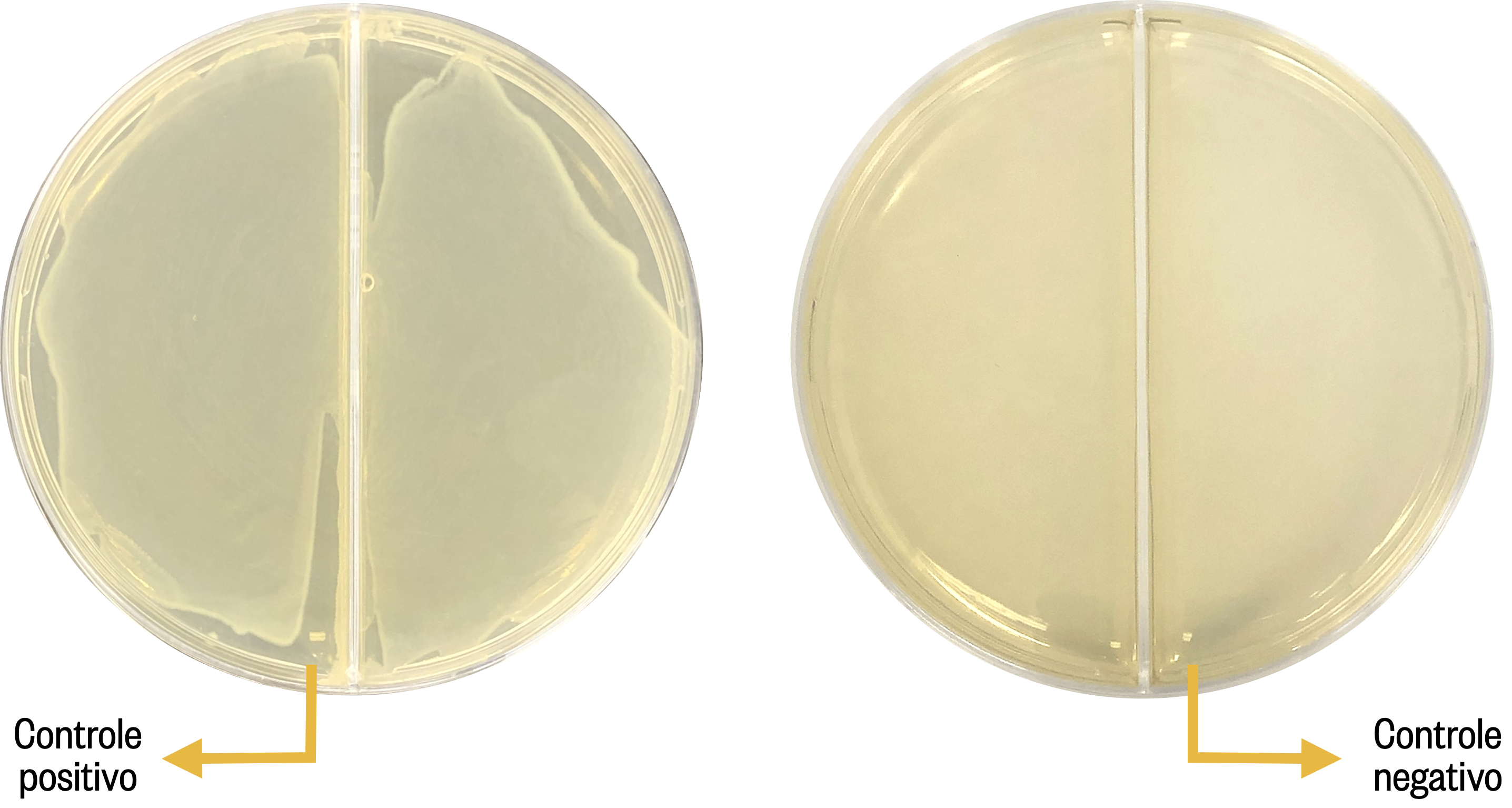


Positive Control

Negative Control


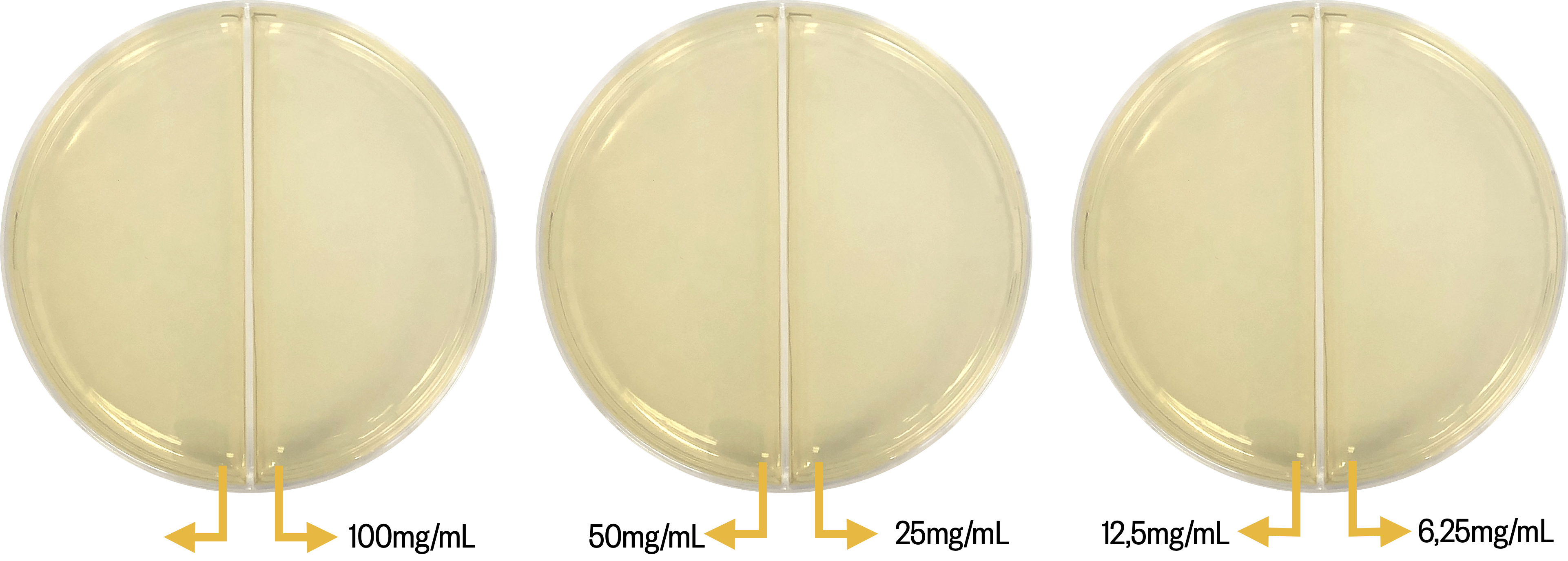


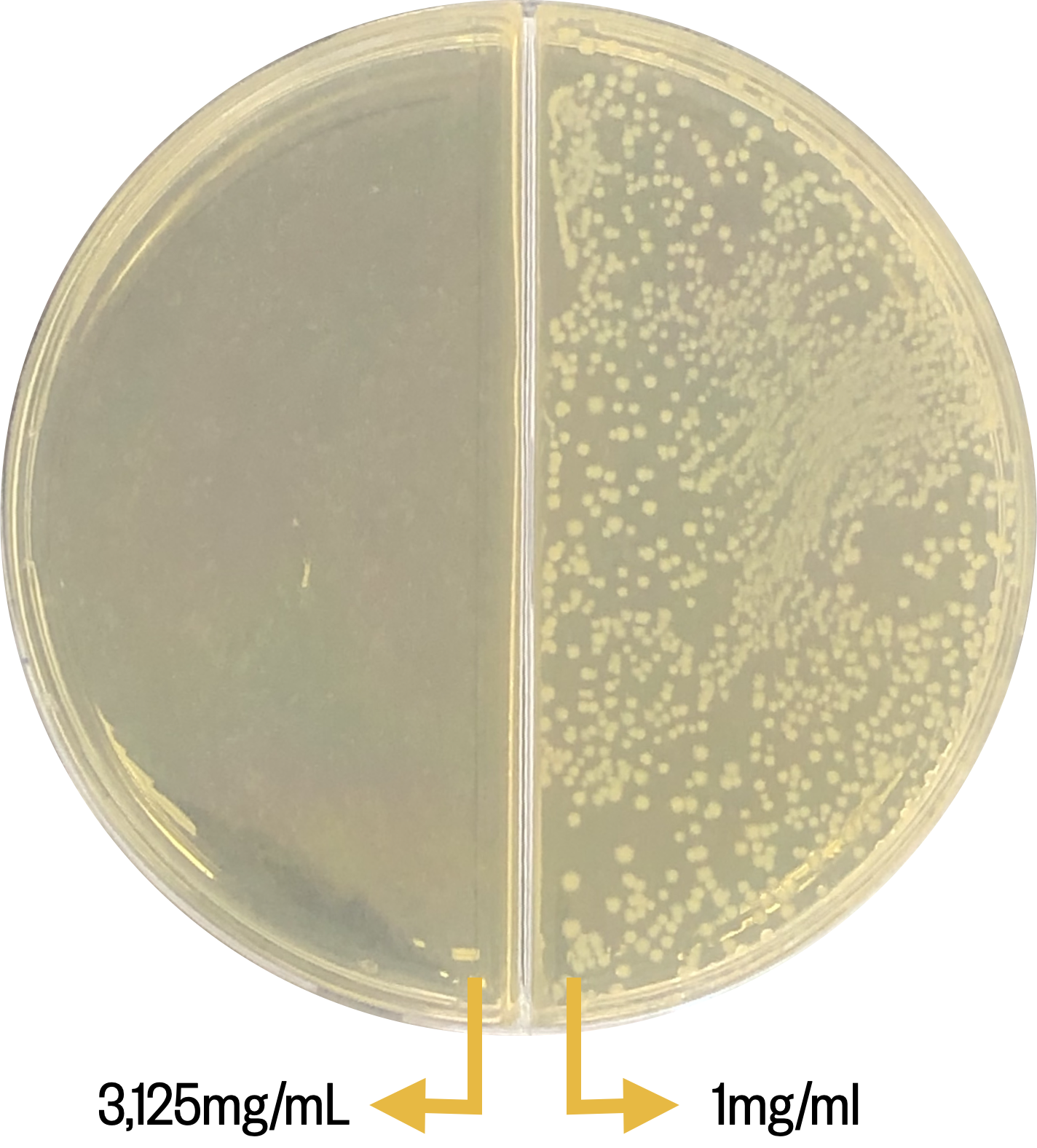

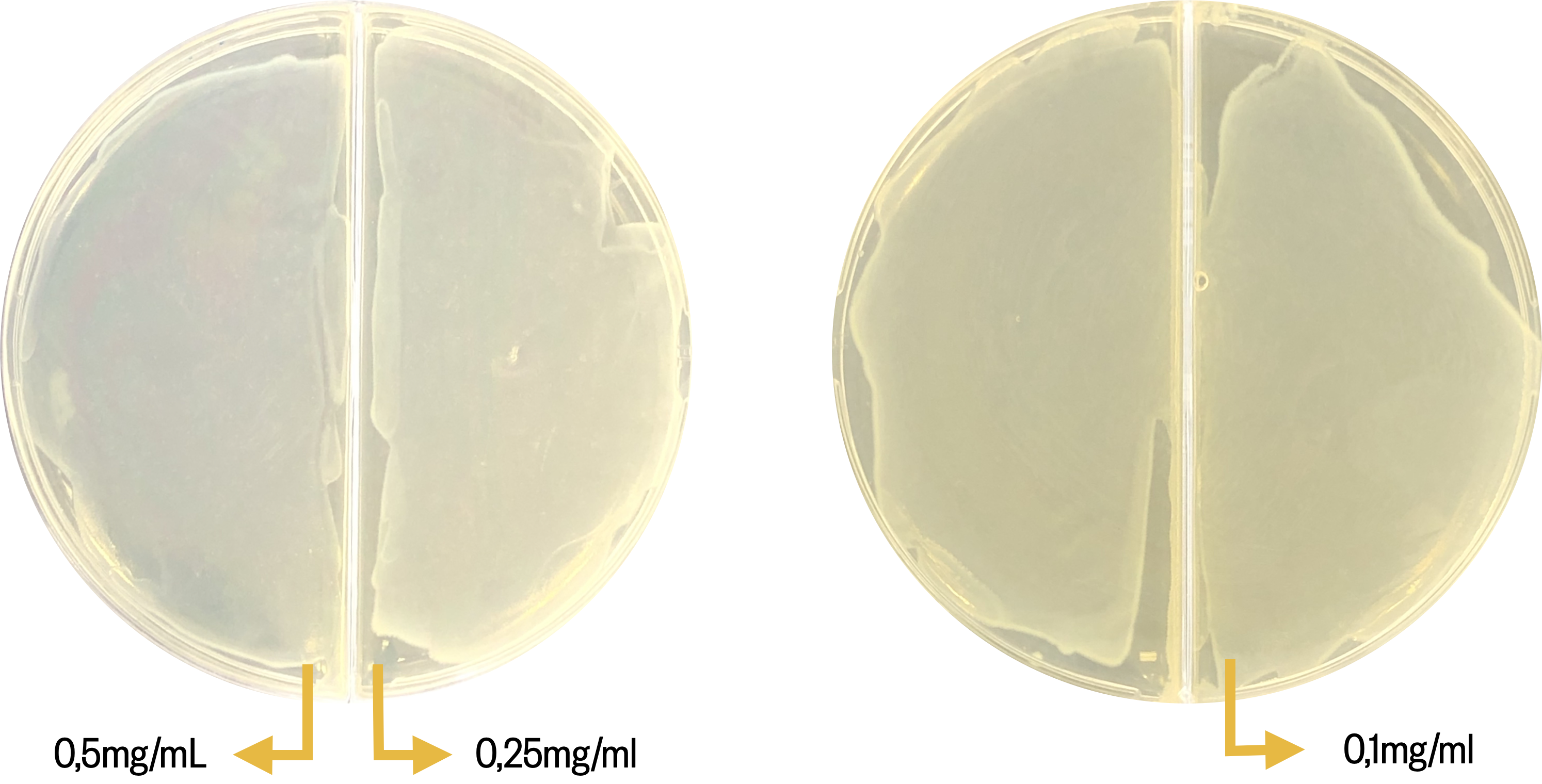


***Figure 1.*** Photograph of Petri dishes illustrating the antimicrobial activity of Ambroxol Hydrochloride at different concentrations.
